# Exploring Therapeutic Targets to Reverse or Prevent the Transition from Metabolically Healthy to Unhealthy Obesity

**DOI:** 10.3390/cells9071596

**Published:** 2020-07-01

**Authors:** Tenzin D. Dagpo, Christopher J. Nolan, Viviane Delghingaro-Augusto

**Affiliations:** 1 Medical School, Australian National University, Canberra, ACT 2601, Australia; tenzin.dagpo@anu.edu.au (T.D.D.); christopher.nolan@anu.edu.au (C.J.N.); 2 Department of Immunology and Infectious Disease, John Curtin School of Medical Research, Australian National University, Canberra, ACT 2601, Australia; 3 Department of Endocrinology, The Canberra Hospital, Garran, ACT 2605, Australia

**Keywords:** obesity, metabolically healthy and unhealthy obesity, adipocyte dysfunction, reversing obesity, therapeutic targets

## Abstract

The prevalence of obesity and obesity-related metabolic comorbidities are rapidly increasing worldwide, placing a huge economic burden on health systems. Excessive nutrient supply combined with reduced physical exercise results in positive energy balance that promotes adipose tissue expansion. However, the metabolic response and pattern of fat accumulation is variable, depending on the individual’s genetic and acquired susceptibility factors. Some develop metabolically healthy obesity (MHO) and are resistant to obesity-associated metabolic diseases for some time, whereas others readily develop metabolically unhealthy obesity (MUO). An unhealthy response to excess fat accumulation could be due to susceptibility intrinsic factors (e.g., increased likelihood of dedifferentiation and/or inflammation), or by pathogenic drivers extrinsic to the adipose tissue (e.g., hyperinsulinemia), or a combination of both. This review outlines the major transcriptional factors and genes associated with adipogenesis and regulation of adipose tissue homeostasis and describes which of these are disrupted in MUO compared to MHO individuals. It also examines the potential role of pathogenic insulin hypersecretion as an extrinsic factor capable of driving the changes in adipose tissue which cause transition from MHO to MUO. On this basis, therapeutic approaches currently available and emerging to prevent and reverse the transition from MHO to MUO transition are reviewed.

## 1. Obesity: A Heterogeneous Disorder of Rapidly Increasing Global Importance

Obesity is a complex condition that occurs due to abnormal or excessive fat accumulation in the body and is diagnosed at a body mass index (BMI) of ≥ 30 kg/m^2^ [1]. The worldwide overweight and obese population has already increased from 857 million in 1980 to over 1.9 billion in 2016 according to the World Health Organization (WHO) estimates, affecting both developed and developing countries [2].

Obesity is a heterogeneous disorder and its pathogenesis involves the interplay between genetic and environmental factors. Mendelian inheritance of severe obesity as a consequence of mutations in genes affecting appetite regulation is rare, such that a polygenic basis is favored to explain the majority of the strong heritability of obesity [3]. Genome-wide association studies (GWAS) have uncovered multiple common variants associated with BMI and obesity with low to modest effect size, the most notable being the fat mass and obesity-associated (*FTO*) gene [3,4]. Environmental factors interacting with a genetically susceptible person are known to contribute, such as ready access to high energy foods (rich in sugars and saturated fats and low in fibers and micronutrients), reduced requirement for physical activity, and even alterations in the gut microbiota [5,6].

With increasing prevalence of overweight and obesity in women of childbearing age, pregnant women are more likely to be overweight or obese and have hyperglycemic disorders such as gestational diabetes and type 2 diabetes (T2D) [7,8,9]. Their offspring are more likely to be born large for gestational age due to fetal hyperinsulinemia and excess adipose tissue accumulation and be at higher risk of childhood obesity, metabolic syndrome (MetS) and T2D [7,8]. Increasing evidence is emerging of the importance of *in-utero* programming through epigenetic processes, such that early life developmental factors combined with genetic risk and childhood and adult environmental exposures add another dimension to the risk of developing obesity [10].

Obesity is associated with increased risks for a plethora of comorbidities. These include cardiometabolic disorders such as non-alcoholic steatohepatitis (NASH), T2D, polycystic ovary syndrome (PCOS) and atherosclerotic cardiovascular disease (ASCVD), as well as disorders not usually classified as cardiometabolic, such as chronic kidney disease, mental health conditions and various types of cancer [11,12,13,14]. Closely associated with all these obesity-related comorbidities are the MetS factors, insulin resistance (IR), hyperinsulinemia, glucose intolerance, hypertriglyceridemia, reduced high-density lipoprotein cholesterol and hypertension [15,16].

In addition to its many comorbidities, obesity is also associated with major economic consequences. For example, individuals with obesity incur medical costs of >30% higher than non-obese individuals. Therefore, obesity is a major cause of concern for governments and healthcare systems worldwide [17]. Thus, development of effective interventions to reduce the incidence of obesity and its related comorbidities are urgently needed, with particular focus on preventing the amplification of this health crisis across generations [10].

## 2. Classification of Obesity as Metabolically Healthy or Unhealthy

At any age, excessive weight gain and obesity are frequently associated with the abnormalities of MetS and increased risk for its associated conditions of NAFLD, PCOS, T2D and ASCVD [15,18]. Interestingly, a subset of individuals within this population (approximately 30%) can be classified as “metabolically healthy obese” (MHO). Although they have excess body weight, they do not display features of MetS, have relatively normal insulin sensitivity and do not have evidence of, or require treatment for, any cardiometabolic diseases [19,20,21]. However, since the definition of MHO has not been fully standardized, variance in its prevalence has been reported [19,21]. Currently, MHO individuals are required to have a BMI ≥ 30 kg m^−2^ or BMI ≥ 25 kg m^−2^ (Asian population) and the absence of the following (or no more than 1 or 2 of them) with abnormal cutoffs that vary according the different criteria for: systolic and diastolic blood pressure (most often ≥130/85), fasting triglyceride (TG) (most often ≥1.7 mmol/L), HDL-cholesterol (most often ≤1.0 mmol/L for men and ≤1.3 mmol/L for women), fasting plasma glucose (most often ≥ 5.6mmol/L) and homeostasis model assessment of IR (HOMA-IR) (multiple cut-offs suggested) or other methods of IR assessment [21,22]. Waist circumference has been included by some, but this makes little sense as all obese persons are likely to have increased waist circumference. It is argued that the allowance of 2 abnormal characteristics, as is used in some criteria, may result in misclassification of MUO as MHO [19]. Additionally, the criteria that can be used for population level studies may need to be different from those used for more advanced clinical research studies, with the latter needing more sophisticated measures of insulin sensitivity, and in some circumstances, liver fat quantification [19]. Clearly, if the terms MHO and MUO are to be used in clinical practice and research, international consensus on definitions is necessary.

Longitudinal and prospective studies have shown that around 50% of individuals with MHO progress to MUO status [22,23,24,25,26]. Increased incidence of cardiovascular events and higher risk for long-term all-cause mortality in the MHO group have also been described [22,23,24,25,26]. Hence, MHO and MUO should not be considered longer-term dichotomous groups, as transition between the two states is possible. Prevention and reversal of the transition of MHO to MUO, therefore, should be considered as therapeutic goals. Thus, understanding the molecular determinants of this transition becomes critical with the potential to also lead into precision medicine approaches. 

In this review, we will examine the main processes involved in adipose tissue development and maintenance, the molecular and biochemical mechanisms at the adipose tissue level that are known to be involved in transition from MHO to MUO status, and the therapeutic interventions currently available to treat MUO. We will also discuss emerging concepts of roles for hyperinsulinemia and disruption of the circadian rhythm in the transition between MHO to MUO that could lead to alternative approaches to obesity management.

## 3. Adipose Tissue Types, Function and Sites

Excess energy is stored as TG in lipid-laden adipocytes in subcutaneous depots of white adipose tissue (WAT), especially in the torso and the proximal regions of the lower and upper limbs (subcutaneous adipose tissue, SAT). These storage areas account for up to 80% of all body fat mass found in humans [27]. The remaining fat (around 10–20% in men and 5–8% in women) is stored in visceral adipose tissue (VAT), which is associated with major neurovascular tracts. VAT is found mainly around internal organs in the body (e.g., heart and kidneys) and in the intra-abdominal area (omental, mesenteric and retroperitoneal fat) [28]. Both depot types are comprised of a heterogeneous cell population constituted of mature adipocytes, supported by a framework of stromal cells, including preadipocytes, fibroblasts, mesenchymal stem cells, vascular endothelial cells and immune cells (macrophages, lymphocytes, dendritic and mast cells) [27]. Brown adipose tissue (BAT) has markedly different morphological and functional characteristics compared to WAT. BAT cells are smaller, contain multiple small lipid droplets and mitochondria, and have richer vascular supply [27]. BAT is capable of very active fatty acid oxidation with heat generation, and for this reason, is important in neonates for body temperature maintenance. BAT is present in adult humans, but in lesser quantities with aging [27]. Beige adipocytes, interspersed within WAT, have cellular characteristics similar to BAT adipocytes, including a high capacity for fatty acid oxidation [29]. “Browning” of WAT is a term used for interventions to increase the number of beige adipocytes [29]. Breast tissue adipocytes transdifferentiate into milk-secreting epithelial cells during lactation [30,31]. Due to their abundant cytoplasmic lipid content, these lipo-secreting epithelial cells have been referred to as “pink adipocytes.” Epithelial-to-adipocyte reversal of this transdifferentiation occurs at the end of the lactation period [30,31]. 

## 4. Healthy and Unhealthy Responses of Adipose Tissue to Excessive Nutrient Supply

Adipose depots are continuously remodeling themselves according to changes in nutritional status. During prolonged periods of food deprivation, they reduce in size, as their lipid content is used to maintain an energy supply to other organs. Conversely, during times of increased nutrient supply, they can rapidly expand in size to enable the safe storage of excess energy as lipid for later use. Of relevance to this discussion, are both the healthy and unhealthy responses of adipose tissue to chronic nutrient supply excess.

Adipose tissue in MUO is characterized by disproportionate accumulation of VAT, adipose inflammation, abnormal adipokine/cytokine production, adipocyte IR and abnormal lipid metabolism. The latter contributes to dyslipidemia and increased lipid accumulation in non-adipose tissues (e.g., ectopic lipid deposition in tissues such as the liver) [32]. Mechanisms proposed for adipocyte dysfunction include hypoxia-induced adipose tissue injury, inflammation, and impaired adipogenesis due to impaired cell number expansion and adipocyte differentiation [33,34,35].

### 4.1. Impaired Adipogenesis and Adipocyte Differentiation Capacity 

**Figure 1:**
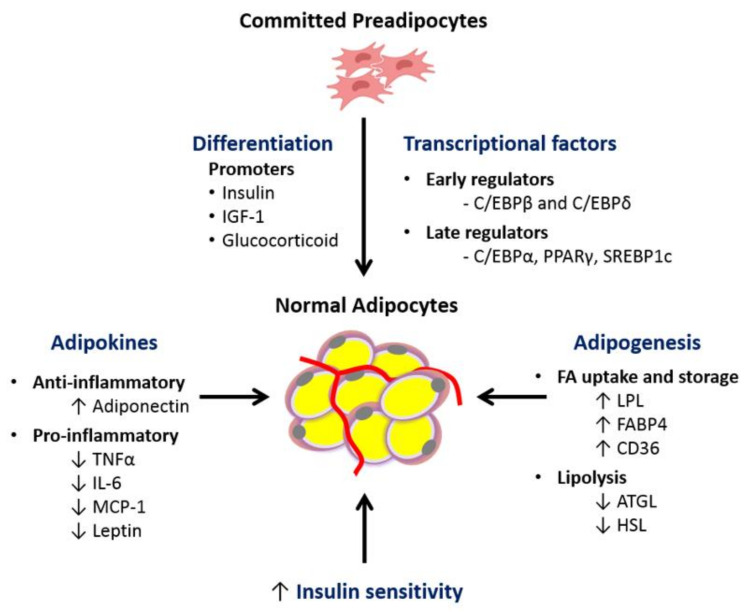
Key transcription factors involved in differentiation of pre-adipocytes to adipocytes that underpin healthy adipocytes. Factors such as insulin, IGF and glucocorticoids can induce differentiation of committed fibroblast-like preadipocytes into spherical adipocytes capable of storing TG within lipid droplets in the cell. Hormonal stimulation triggers differentiation by the expression of early differentiation factors C/EBP-β/δ and late transcription factors C/EBP-α, PPAR-γ and SREBP-1c. PPAR-γ is known as the main regulator of adipogenesis and modulates the expression of various genes associated with fatty acid uptake, storage, lipolysis and adipokines. (IGF, insulin-like growth factor; C/EBP, CCAAT/enhancer binding protein; PPAR-γ, peroxisomal proliferator-activated receptor gamma; SREBP, Sterol regulatory-element binding protein; FA, fatty acid; TNF-α, tumor necrosis factor alpha; IL-6, interleukin 6; MCP-1, monocyte chemoattractant protein-1; LPL, lipoprotein lipase; FABP4, fatty acid binding protein 4; ATGL, adipose triglyceride lipase; HSL, hormone sensitive lipase).

Hyperplasia is an alternative process to hypertrophy for adipose tissue expansion [36,37,38]. This process comprises the proliferation and differentiation of fibroblast-like preadipocytes into mature lipid-laden adipocytes. It is a complex and dynamic process regulated by several transcription factors and cell cycle proteins [39]. The initial phase of adipogenesis, requires the conversion of pluripotent mesenchymal stem cells to committed preadipocytes, which are morphologically similar but restricted to differentiation into adipocytes [38]. In vitro studies have shown that upon hormonal stimulation, committed preadipocytes undergo differentiation triggered by the expression of early CCAAT/enhancer binding protein (C/EBP) β and δ and late transcriptional factors peroxisome proliferator activated receptor-γ (PPAR-γ) and C/EBP-α [36]. It culminates with mitotic clonal expansion and changes in cell morphology, from a fibroblast-like shape to a spherical shape, followed by the functional ability to accumulate lipid droplets [36] (Figure 1).

Cumulative evidence showing impaired replicative and adipogenic capacity of different fat depots have been described in subjects with lipodystrophy, obesity-related IR and morbid obesity [40,41,42,43]. Consistent with this, when adipocyte size is sorted based on size distribution instead of average size, elevated frequency of very small SAT adipocytes and reduced numbers of larger adipocytes were reported in overweight subjects with IR and T2D [41,44,45]. However, other studies have also shown increased mean adipocyte size, particularly in VAT, in obese subjects with abnormal metabolic phenotype [46,47,48].

Increasing evidence points to reduced expression levels of key adipocyte differentiation transcription factors in adipose tissue from MUO individuals. PPAR-γ is considered a major regulator of adipogenesis as it plays a central role in the maintenance of mature adipocyte function and insulin sensitivity [49,50]. It activates genes involved in fatty acid uptake and storage, such as adipose protein-2 (*AP2*)/fatty acid binding protein-4 (*FABP4*), lipoprotein lipase (*LPL*) and acyl CoA synthase (*ACS*) [36]. PPAR-γ also targets adiponectin, a hormone produced by mature adipocytes that promotes fatty acid oxidation and insulin sensitivity [51]. Several studies have suggested that the transcription factor sterol regulatory element binding protein-1c (SREBP-1c) promotes PPAR-γ activity by producing lipid moieties that are endogenous PPAR-γ ligands [52]. Reduced expression of SREBP-1c and PPAR-γ in WAT of obese, insulin-resistant individuals has been described compared to insulin-sensitive controls, suggesting possible defects in adipocyte differentiation [40,41,42].

### 4.2. Hypoxia-Induced Adipose Tissue Injury and Adipose Tissue Inflammation

It is well-known that in conditions of short-period nutrient excess, SAT adipocytes become hypertrophic and are briefly exposed to hypoxia due to inadequate vascularization. This acute effect triggers a stress response that promotes angiogenesis and remodeling of the extracellular matrix, restoring a healthy oxygen supply to the expanding adipose tissue [53]. Several studies have suggested that inadequate vascularization results in adipocyte dysfunction including cell death, recruitment of inflammatory cells including M1 macrophages that form “crown-like structures” around the dead cells, increased cytokine production and altered adipokine release [54]. This process causes inappropriate extracellular matrix remodeling and fibrosis, consequently restricting the lipid storage capacity of adipose tissue, causing lipid spillover into circulation and ectopic fat accumulation [55].

Although the mechanisms involved are not fully understood, hypoxia is known to increase adipose expression of the transcriptional factor hypoxia inducible factor-1α (HIF-1α), which can induce inflammation, fibrosis and IR in rodent WAT [56,57,58]. Possibly related are the recently observed associations of CD248 with adipose tissue hypoxia, inflammation, and fibrosis [59]. CD248 is a sensing transmembrane glycoprotein that is highly expressed in WAT of individuals with obesity, IR and diabetes, and reduced after bariatric surgery [59]. In human adipocytes, *in vitro* knock down of *CD248* by siRNA attenuated hypoxia-induced HIF-1α promoter activity, as well as the expression of a whole cluster of hypoxia-induced genes. Furthermore, adipocyte-specific *CD248* knockout in mice protected against high fat diet-induced insulin resistance, glucose intolerance and WAT dysfunction, the latter evidenced by increased vascularization, reduced HIF-1α, reduced macrophage infiltration, and reduced markers of fibrosis [59]. Therefore, adipose vascularization, its extracellular matrix composition, and *CD248* could be targets for the prevention of adipose tissue transition to MUO.

Taken together, the inability to recruit and/or to differentiate more preadipocytes into functional adipocytes in conditions of positive nutrient imbalance, could be playing a role in transition from MHO to MUO status. Therefore, the use of therapeutics agents that promote adipogenesis could improve adipocyte lipid storage capacity, and consequently, avoid transition to MUO.

### 4.3. Altered Adipokine/Cytokine Production

Healthy adipose tissue secretes a wide range of adipokines, which not only modulate biological processes within the adipose tissue, but also exert regulatory functions in other organs, including the liver, pancreas, muscle and brain, as well as in vascular and immune system cells/tissues. The most commonly quantified adipokines in human studies include adiponectin and leptin [60].

Adiponectin is found in high levels in the circulation of healthy individuals, but is decreased in subjects with obesity, T2D and NAFLD [61,62] (Figure 1). Adiponectin is an insulin sensitizing adipokine via its capacity to activate AMP-activated protein kinase, which supports enhanced fatty acid oxidation and glucose utilization in skeletal muscle and the suppression of gluconeogenesis within the liver [63,64]. It also has anti-inflammatory properties, since it suppresses TNF-α production and stimulates synthesis of anti-inflammatory IL-10 [65]. In contrast, leptin, whose circulating levels are proportional to body fat mass, is involved in appetite control and energy expenditure. High levels of leptin should prevent overfeeding through suppression of orexigenic peptides and stimulation of anorexigenic peptides in the central nervous system (mainly in the hypothalamic area). However, most obese subjects develop resistance to its action [66,67]. Chronically elevated leptin levels are pro-inflammatory, increasing the production of cytokines such as TNF-α, IL-6 and monocyte chemotactic protein-1 (MCP-1) from tissue-resident immune cells [68]. Serum adiponectin levels are reduced in MUO compared to MHO, whereas no difference in leptin is generally observed [69,70].

Other adipokines, such as chemerin and osteonectin, have also been investigated [71]. Chemerin and its receptor CMKLR1 are highly expressed in mature adipocytes and play regulatory roles in adipogenesis, adipocyte metabolism and recruitment of immune cells to inflammatory sites [71,72]. Cross-sectional studies reported increased levels of circulating chemerin in children and adults with obesity. However, when subjects are stratified into MHO and MUO, both no change and elevated levels of chemerin have been reported in MUO [70,73,74]. Osteonectin, an adipokine involved in the regulation of extracellular matrix composition and inhibition of adipogenesis, was recently assessed within a large pediatric Chinese cohort and it was found to be augmented in the circulation of children with MUO [75].

The mix of cytokines produced by adipose tissue (adipocytes and resident immune cells) is inflammatory in obese subjects, with evidence of greater effect in MUO. TNF-α, IL-6 and MCP-1 interfere with hepatic insulin signaling through inhibition of IRS1 phosphorylation [76,77]. Additionally, TNF-α augments adipocyte lipolysis, IL-6 suppresses adiponectin expression and MCP-1 increases hepatic TG accumulation [51,77,78,79]. Conflicting results have been reported in human studies comparing MHO and MUO, where either no difference or increased serum TNF-α and IL-6 in MUO were found [69,80,81].

There are still limited number of studies comparing adipokine expression levels in MHO and MUO with metabolically normal lean subjects. Of these, differences in race, age, gender, source of sample collection, the definition of MHO used, as well as small number of participants, have contributed to inconsistent results limiting their usefulness in the clinical setting.

### 4.4. Dysfunctional Adipose Tissue Lipid Metabolism

MUO compared to MHO individuals display dyslipidemia and ectopic fat deposition in many organs of the body including liver, muscle, heart and pancreas [21,82].

Healthy adipose tissue is insulin sensitive, such that at times of increased nutrient supply and in response to meal-stimulated insulin secretion and insulin action, it can readily store excess energy safely as TG in lipid droplets [83,84]. Postprandial lipid storage in adipocytes is initiated with the uptake of free fatty acid (FFA) released from circulating TG-rich lipoproteins (chylomicrons and very low density lipoproteins (VLDLs)), by the action of lipoprotein lipase (LPL) with activation by insulin [85,86]. LPL is found on the luminal side of the capillary endothelium associated with glycosaminoglycan, and is considered the main gatekeeper for the entry of FFA from circulating TG into adipocytes, where it is re-esterified into glycerolipids, particularly TG, for storage [87]. Its transcription is initiated by the action of SREBP-1c and PPAR-γ and its action is promoted by insulin; whereas its inhibition is promoted by tumor necrosis factor alpha (TNF-α) [88,89]. Following LPL action, FFA are transported into adipocytes by the integral membrane protein CD36/fatty acid translocase (CD36) and by the cytoplasmic protein FABP4/aP2 [87]. During periods of energy deficit, at times of low serum insulin/glucagon ratio, fasting and exercise, the intracellular lipolytic enzymes adipocyte triglyceride lipase (ATGL), hormone sensitive lipase (HSL) and monoacylglycerol lipase (MAGL) hydrolyze TG stored in the lipid droplets, releasing FFA and glycerol to meet the energy demands of vital organs [90].

In MUO obesity, these processes of adipose tissue lipid storage and release become dysfunctional as a consequence of IR and adipose tissue inflammation [91,92]. Triglyceride lowering by insulin has been shown to be inversely related to the level of IR [93]. Obese patients with both MHO and MUO have reduced gene expression of factors that promote lipid uptake and processing in comparison with healthy lean persons [94,95]. In multivariate regression analysis, increasing BMI was most strongly associated with reduced *LPL* and *FABP4* gene expression in both SAT and VAT, within subjects with a spectrum of low to high IR [96]. Adipose tissue IR results in dysregulated lipolysis of stored TG [42]. Although limited information is available about the expression levels of lipase enzymes in human adipose tissues of MHO and MUO, increased mRNA expression of *ATGL* and *HSL*, in both VAT and SAT, have been described in conditions of morbid obesity with variable degrees of IR [42].

**Figure 2:**
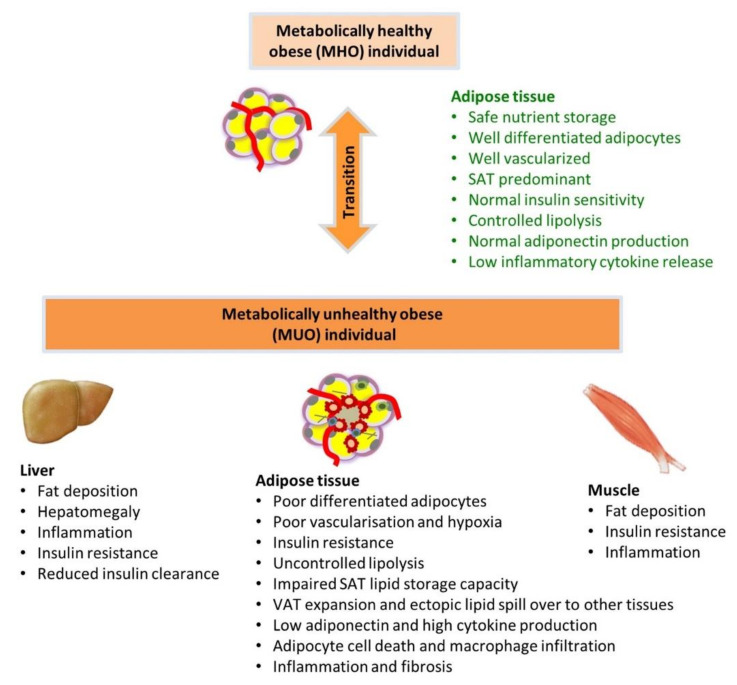
Characteristics of adipose tissue in metabolically healthy obesity (MHO) and metabolically unhealthy obesity (MUO). In MHO individuals, adipose tissue is capable of expansion to enable safe storage of excess energy as lipids in subcutaneous adipose tissue (SAT) depots. However, in individuals who transition to MUO, there is restriction in further SAT expansion, resulting in disproportionate lipid accumulation in visceral adipose tissue (VAT), as well as spill-over of lipid from adipose tissue into circulation and to ectopic deposition in other tissues such as liver and skeletal muscles. Adipose tissue in MUO is also characterized by adipose inflammation, abnormal adipokine/cytokine production, adipocyte insulin resistance and abnormal intracellular lipid metabolism, which underlie the dyslipidemia and systemic inflammation of the MUO state. (SAT, subcutaneous adipose tissue; VAT, visceral adipose tissue).

Thus, dysfunctional transport of FFA into adipocytes related to IR, as well as impaired suppression of breakdown of stored TG by insulin, contribute to the development of dyslipidemia and ectopic lipid deposition and tissue injury, characteristic of MUO (Figure 2).

### 4.5. Role of Epigenetics

The epigenome, which comprises chemical modifications within the DNA (e.g., methylation marks) and closely associated molecules (e.g., histones), determines which genes within a cell are expressed. Much of the epigenome is determined through development and is influenced by early life environment, but some aspects are clearly modifiable by later life environment [97,98]. Key to this discussion is the role of the epigenome in determining the behavior of adipose tissue cells in the transition between MHO and MUO. There are studies of human adipose tissue that do indicate associations of epigenetic changes in adipose tissue with obesity. Furthermore, interventions during adult life have been shown to induce epigenetic changes within this tissue [99,100,101,102,103].

Indicative of epigenomic change associated with obesity, a study conducted within an adult European discovery cohort, and confirmed in two separate validation cohorts, reported increased methylation within three separate sites in intron 1 of the *HIF3A* locus in adipose tissue associated with increased BMI [102]. Both exercise and bariatric surgery have shown to have effects on DNA methylation status within adipose tissue, consistent with the potential for modifying the epigenome of fat through interventions [100,101]. Relevant to the epigenome and transition between MHO and MUO, Crujeiras et al. compared the global methylome within VAT of IR and insulin sensitive morbidly obese individuals [103]. They found DNA methylation was altered within the VAT of IR in 982 CpG sites encoding 538 unique genes. The identified genes were involved in functions such as cell adhesion, collagen-related, signal transduction and transcriptional regulation [103].

These studies support a role of epigenetics in MHO to MUO conversion, but the small numbers of subjects limits interpretation and further larger studies are required.

## 5. Factors Extrinsic to Adipose Tissue Contributing to MUO

**Figure 3:**
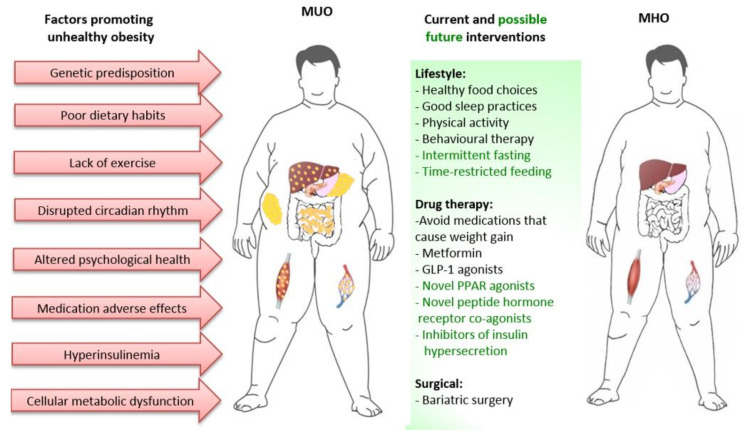
**Figure 3**. Current and possible future interventions to prevent and reverse transition of MHO to MUO. Several factors are involved in the development of metabolic unhealthy obesity (left panel). Therefore, to prevent or reverse transition of MUO to MHO (right panel) it is necessary to adopt healthy lifestyle changes. However, lifestyle changes alone will be insufficient for many, such that combination with drugs that directly reverse metabolic abnormalities of MUO and/or promote substantial weight loss, or bariatric surgery is likely to be also required. (MUO, metabolically unhealthy obesity; MHO, metabolically healthy obesity; GLP-1, glucagon-like-peptide 1; PPAR, peroxisomal proliferator-activated receptor).

Adipose tissue characteristics clearly differ between individuals with MHO and MUO, as already discussed. However, the extent to which the transition from MHO to MUO is due to factors intrinsic or extrinsic to adipose tissue is less clear. These factors include periods of greater nutrient excess, alterations in the gut microbiome, disruptions of the circadian rhythm affecting neuro-hormonal systems, pancreatic islet dysfunction resulting in hyperinsulinemia, metabolic dysfunction of other tissues such as muscle and liver, psychological health and adverse effects of some medications [15,104,105,106,107]. This is important, as approaches/therapies to prevent or reverse progression from MHO to MUO may need to be multifaceted, with focus on direct modification of adipose tissue as well as on factors extrinsic to adipose tissue (Figure 3).

### 5.1. Total Body Energy Balance, Pattern of Eating, Nutrient Quality, and Exercise

In an obese person, total body energy balance can vary over time as a consequence of dietary/lifestyle programs, psychological factors, co-morbidities affecting capacity to exercise and psychotropic medications [106,107,108]. There is considerable evidence that periods of excess energy balance as opposed to negative energy balance, will worsen the MetS factors that underpin the diagnosis of MUO [109,110]. Modest weight loss does reverse the presence of MetS factors in obese subjects and result in improvements in adipocyte function [111,112,113]. Marked weight loss as is possible with bariatric surgery, also improves metabolic health. For example, the Roux-en-Y gastric bypass (RYGB) operation, which promotes weight loss through both restriction of nutrient intake and malabsorption, results in sustainable weight loss, improved metabolic profile (reductions in total cholesterol, LDL-c, TG, hepatic enzymes), ameliorated inflammatory status (reduced CRP, TNF-α and IL-6) and elevated adiponectin and incretin hormones [114,115,116]. Thus, prevention of weight gain and promotion of weight loss is key in preventing MUO.

Recently, there has been particular interest on the effects of altering eating patterns to improve metabolic health, through programs of intermittent fasting, periodic fasting and time restricted feeding, with the view that this will improve metabolic flexibility, metabolic health and longevity, with at least part of this effect being independent of weight loss [117,118]. Human studies of altered eating patterns are of relatively short duration and suggest benefit. However, the capability of obese individuals to sustain such approaches in the longer term is unknown and outcomes of further studies are required [119] (Figure 3). 

The composition of diet is also important with evidence suggesting that higher dietary carbohydrate intake, in particular, high intake of sugar sweetened beverages is associated with greater risk of developing MetS [120,121]. However, low carbohydrate diets associated with increased saturated fat may have detrimental effects on lipid parameters. The Mediterranean diet in which the quality of sourcing of macronutrients (e.g., vegetables, salads, fruit, olive oil, fish) is most important is gaining favor [122]. 

Additionally, dietary habits can affect the diversity, composition, and stability of the gut microbiome, with increasing evidence that it can contribute favorably or unfavorably to maintenance of normal metabolic homeostasis and body weight [6,123,124]. Haro et al. have demonstrated that long-term consumption of Mediterranean or low-fat, high-complex carbohydrate diets increase the abundance of diabetes-protective bacterial species, therefore, resulting in better insulin sensitivity in those at risk [125]. Furthermore, significant fat mass loss after bariatric surgery in MUO individuals has been linked to augmented abundance of Proteobacteria and better metabolic health [126].

Exercise will assist in achieving negative energy balance and weight loss, but also will increase general fitness, prevent sarcopenia, and reverse MetS factors independent of weight loss [127,128,129,130]. An additional mechanism of benefit from exercise may be via exercise-induced release of a variety of beneficial myokines, such as IL-6, which increases the circulating levels of cytokines IL-10 and IL-1ra [131,132]. IL-6 also inhibits the production of TNF-α, thus promoting an anti-inflammatory effect [133]. Other exercise-induced myokines, such as irisin and myostatin, are reported to be involved in crosstalk between skeletal muscle and adipose tissue, with potential to have browning effects on WAT [134,135], but require further investigation with respect to their importance in human MHO and MUO [136,137,138]. The benefit on MetS factors of combining exercise, caloric restriction and the Mediterranean diet was shown after the first year of the PREDIMED-plus study [139].

### 5.2. Disruptions in the Circadian Rhythm

The daily timing of waking, eating meals, physical activity, body temperature and sleeping should have a regular pattern, with regulation by central and peripheral clocks and the neuro-hormonal systems of the body [104,105,117]. Normal metabolic functions are tightly linked to this circadian rhythm, with high capacity of the body to transition rapidly from the fasted to the fed state, a process that has been described as “metabolic flexibility” [117]. MetS is associated with loss of metabolic flexibility such that it has even been suggested that MetS be named the ‘circadian syndrome” [105]. Circadian rhythm disturbances are likely to be the consequence of the modern-day environment [104,105,140]. For example, light pollution at night within cities and the greater individual use of electronic screen devices into the night are proposed to be major contributors to the dysregulation [140,141]. Night shift-work rostering is known to be strongly associated with overweight, obesity, MetS and MetS-related diseases [142]. Furthermore, multidirectional causality between disturbances in circadian rhythm, psychological disorders, and some medications of mental illness, overweight/obesity and MetS have been suggested [104,106]. Therefore, interventions that include focus on circadian aspects of lifestyle in prevention of conversion from MHO to MHO should be considered.

### 5.3. Potential Role of Hyperinsulinemia as Driver to MUO Phenotype

In recent years, the notion of insulin being the initial factor causing obesity and its metabolic comorbidities has re-emerged [15]. Increasing evidence from pre-clinical and clinical studies support a view, at least in subsets of at-risk individuals, that hyper-responsiveness of the islet β-cell to a hostile environment (e.g., from a westernised lifestyle) drives hyperinsulinemia, and the hyperinsulinemia is upstream to excessive weight gain and MetS, including the development of IR [15,143,144,145,146]. In mouse models, various genetic manipulation approaches to attenuate insulin secretion protects the mice from diet-induced obesity, IR and hyperglycemia [147,148].

Of relevance within human studies, is the Da Qing Children Cohort Study which showed that fasting insulin at about 5 years of age, after the adjustment for age, sex, birth weight, TV-viewing time and weight (or body mass index) at baseline, predicted weight gain from age 5 to 10 years [149]. Furthermore, higher insulin levels at 5 years of age were also predictive of higher levels of systolic blood pressure, fasting plasma glucose, IR as determined by the homeostasis model, and TG at 10 years of age, all features of MetS [149]. The findings were similar to those in a study of Pima Indian children [150]. Pharmacological approaches with diazoxide or the somatostatin analogue octreotide-LAR to suppress insulin secretion in humans, also support the view that hyperinsulinemia may have more of a primary role in MetS [151,152]. Thus, therapies aimed to reduce insulin hypersecretion in obese subjects, particularly in its early stages of development, may have the potential to prevent progression to MUO.

### 5.4. Altered Whole Body Amino Acid Metabolism

Metabolomic and transcriptomic analysis of plasma samples also reveal distinct amino acid profiles between lean, MHO and MUO groups, suggesting a potential interplay between amino acid metabolism, obesity and metabolic status [153,154]. Branched-chain amino acids (BCAA) valine, isoleucine and leucine were increased in MHO and MUO compared to lean groups, with MUO tending to have higher levels than MHO [153]. Positive associations between the BCAA isoleucine and IR and HbA1c have also been described, indicating possible use of BCAA as markers for early identification of IR [154]. Reduced levels of glycine, an indicator of increased gluconeogenesis and IR, were also observed in MUO [153,154]. Tyrosine and phenylalanine aromatic amino acids were elevated in both MHO and MUO compared to lean subjects [153]. While multiple tissues including gut, liver, skeletal muscle and kidney are clearly involved in amino acid metabolism, transcriptome analysis of adipose tissue obtained from these groups showed impaired expression of several genes associated with BCAA catabolism [154]. It is unclear whether the amino acid changes predictive of moving between the states of MHO to MUO are pathogenically involved in this transition, as this would provide support for approaches to manipulate their levels in prevention strategies.

## 6. Approaches to Prevent or Reverse the Progression from MHO to MUO

As discussed above, transition between MHO and MUO is possible, such that prevention and reversal of progression from MHO to MUO should be a therapeutic goal [23,155,156]. Identifying those at greatest risk of progression needs to be considered, as focus of more intensive intervention efforts on these individuals will likely be rewarding. Therapeutic approaches can be divided into those that prevent or reverse progression with no or minimal weight loss, and those that are directed at achieving major weight loss. For all approaches, it is important to not consider each therapy in isolation, as combining therapies to gain synergism of actions is likely to provide greater benefit.

### 6.1. Predicting Subjects at Risk of Progression to MUO

Within a Japanese population, greater visceral adipose area as measured by computerized tomography, together with lower levels of HDL-cholesterol, higher plasma insulin and female sex predicted progression from MHO to MUO over a 10-year period [156]. Similarly, within a Korean population, a visceral adiposity index derived from BMI, waist circumference, HDL-cholesterol and plasma TG also predicted this progression [157]. Of blood biomarkers, uric acid was an independent variable that could predict progression [157]. Further development of risk prediction tools of progression between MHO to MUO taking into account ethnic differences, is likely to be worthwhile.

### 6.2. Lifestyle Interventions with no or Minimal Weight Loss

The evidence suggests that dietary and exercise measures that affect some minimal weight loss are beneficial. For example, Magkos et al. reported that a 5% lifestyle-induced body weight reduction has some beneficial metabolic outcomes of improved insulin sensitivity and islet β-cell function; but 10-15% weight reduction has additional benefits, such as reduction in hepatic steatosis and in adipose tissue expression of genes involved in oxidative stress and extracellular matrix production [111]. Meta analyses of lifestyle studies in the management of NAFLD and PCOS are consistent with the beneficial effects of healthy diet and exercise that affect at least some weight loss [158,159].

It is unclear whether a particular diet or exercise program, assuming equivalent amount of weight loss, is superior to others. Considering the potential role of hyperinsulinemia in progression of MHO to MUO, meal plans associated with lower post-prandial insulin increments should potentially be of advantage. This may underlie movement towards lower content of sugars and carbohydrate in general and avoidance of Western dietary patterns [15,160,161]. This may also be a factor contributing to the success of the Mediterranean diet in MetS and NAFLD [162]. Additionally, the pattern of food intake may also be an important means to reduce insulin levels. The prospective role for intermittent fasting and time-restricted eating on preventing MHO progressing to MUO needs to be further investigated, as these approaches have been shown to reduce basal insulin secretion [163,164].

The potential impact of improving sleep hygiene to normalize circadian rhythm, in order to lessen MetS features and prevent progression to MUO, deserves attention [105,106]. Improved use of psychotropic medications linked to weight gain should also be a priority.

### 6.3. Pharmaceutical Interventions with no or Minimal Weight Loss

The biguanide metformin, which is used as a first line glucose-lowering agent in T2D, may have a role in preventing progression to MUO. In the long-term follow up of the Diabetes Prevention Program, metformin reduced T2D incidence rate in high risk individuals by 17–36% (using glucose and HbA1c criteria, respectively) [165]. In PCOS, metformin has also been shown to be of benefit, but with minimal effects on liver abnormalities in NAFLD [162,166]. It is safe and could be particularly valuable when used in combination with other therapies, as is often the approach with metformin in treatment of T2D.

PPAR-α, β/δ and γ agonists affect transcription of metabolic genes in adipose and other tissues and have been extensively investigated in MetS related conditions [167,168]. The thiazolidinediones (TZDs) are PPAR-γ agonists that are well known to be whole body insulin sensitizers. TZDs have potent direct beneficial effects on adipose tissue, promoting adipocyte differentiation, insulin sensitivity, lipid storage capacity and adiponectin production, and lowering the release of inflammatory cytokines at both local and systemic inflammation [168,169]. Fibrates are PPARα agonists used for their capacity to lessen the dyslipidemia of MetS characterized by elevated TG and reduced HDL-cholesterol levels [167,168]. They are known activators of fatty acid oxidation pathways in tissues such as liver, heart, skeletal muscle and BAT [167,168]. Activation of PPAR-β/δ also activate fatty acid oxidation, predominantly in skeletal muscle. There are currently no PPAR-β/δ agonists used in clinical practice. Therefore, PPAR receptor agonists have enormous potential in reversing multiple components of MetS, whether used as singular, dual or pan agonists or selective PPAR modulators (SPPARMs). However, many candidate agents have failed due to adverse side effect profiles [167,168]. The TZD agents rosiglitazone and pioglitazone were highly favorable glucose lowering drugs, but their use has been dramatically curtailed due to issues with weight gain, fluid retention, heart failure, bone fractures and possible increases in cardiovascular event risk (rosiglitazone) and bladder cancer (pioglitazone) [170]. There is still some support for using pioglitazone in T2D for cardiovascular protection, but the adverse side effects make it less attractive for prevention of MUO [169,170].

Some new PPAR agonist agents do show promise. The SPPARMα agent pemafibrate, which is in clinical use in Japan, is more potent than fenafibrate at reversing the atherogenic dyslipidemia of MetS and T2D, and shows promise in being beneficial in NAFLD, without evidence of liver or renal toxicity [171,172]. A dual PPAR-α/γ agonist saroglitazar is in clinical use in India for diabetic dyslipidemia. Saroglitazar has been shown to have beneficial effects on TG and HDL-cholesterol levels as well as HbA1c, without the issues of weight gain and fluid accumulation as occurs with the singular PPAR-γ agonists [173,174].

### 6.4. Pharmaceutical Interventions with greater effect on Weight Loss

As positive energy balance is associated with worsening of MetS, therapies that achieve substantial negative energy balance should be beneficial [109,110]. The glucagon-like-peptide 1 (GLP-1) agonists have an established role in management of T2D, with evidence of reduced occurrence of major cardiovascular events and death [175]. GLP-1 agonists also have an established role in obesity due to their effects on appetite and food intake from their actions in the gut to delay gastric emptying and in the brain [176]. In small studies, the GLP-1 receptor agonist liraglutide has been shown to have beneficial effects in both NAFLD and PCOS [177,178]. The beneficial effects may be secondary to weight loss rather than direct effects on insulin sensitivity and cellular metabolic pathways. This is consistent with the results of a study of liraglutide in obese adolescents in which weight loss was achieved without significant change in other MetS parameters, such as insulin sensitivity and lipid levels [179]. The GLP-1 receptor agonists are safe but associated with significant symptoms of nausea and vomiting in some subjects [176].

In recent years, technological advances in drug discovery has led to the development of a series of peptide hormone receptor co-agonists, including several GLP-1/glucagon and GLP-1/gastrointestinal insulinotropic polypeptide (GIP) receptor co-agonists, that have potent anti-obesity effects and are in various phases of clinical trials [180]. An example of such agents progressing through clinical trials is the GLP-1/GIP receptor co-agonist tirzepatide (LY3298176), which was found in a phase-two study to result in greater weight loss and improvements in glycemic control than the GLP-1 agonist dulaglutide, with acceptable safety and tolerability [181]. In a post-hoc analysis of this trial, tirzepatide was shown to be beneficial on biomarkers of nonalcoholic steatohepatitis (NASH) and fibrosis [182]. The anti-obesity drug development pipeline focuses on multiple targets, including those that act by increasing metabolic rate, such as via the fibroblast growth factor 21 (FGF21) signaling pathway and browning of adipose tissue [183]. Many will have the potential to prevent and reverse progression from MHO to MUO, but it is important to consider the safety and cost benefit of these agents.

### 6.5. Bariatric Surgery

Bariatric surgery continues to be the most effective means to achieve substantial sustainable weight loss and should be considered in the management of individuals with MUO. As already discussed, it is capable of reversing MetS characteristics and for this reason should also be effective in preventing and reversing progression of MHO to MUO [116,184]. Of note, within only one week following RYGB an impressive reduction in fasting insulin levels is observed prior to changes in body mass [185].

### 6.6. Preventing Insulin Hypersecretion

All therapies that are successful in reversing of MetS can also lower insulin levels and improve insulin sensitivity. This raises the question as to whether more focus of therapies to prevent MetS and MUO, should be on their effects on hyperinsulinemia, either through lifestyle measures or pharmacological treatments. Further to this, considering the possible up-stream role that hyperinsulinemia could be playing in MetS, treatments that reduce insulin secretion more directly may be beneficial, particularly early in obesity before it progresses to MUO [15,145,146,186].

## 7. Conclusions

Preventing and reversing the transition between MHO into MUO is a worthy target for reducing the burden of obesity. Understanding the underlying pathophysiological mechanisms is clearly important, with these mechanisms being both intrinsic and extrinsic to the adipocyte. The re-emergence of the concept that pancreatic beta-cells’ insulin hyper-responsiveness to nutrient-stimulus might be the factor triggering obesity, warrants further investigation. On a population basis, improving lifestyle factors related to diet, exercise and sleep health should be a priority. For overweight and obese individuals, combining lifestyle measures with pharmaceutical therapies, looking for synergism in effects should be the goal. There are exciting new drug treatments on the horizon that are either highly effective at weight loss and/or at reversing defects of MUO at the cellular level. However, there is much more work required to ensure safety. This is particularly important if these drugs are to be used in relatively young people with obesity. While waiting for some of the highly potent weight loss drugs to be deemed safe, bariatric surgery will remain the most effective treatment for those individuals at highest risk of MUO progression.

A major limitation in moving forward is the lack of internationally accepted definitions of MHO and MUO, including practical diagnostic criteria, that does not allow accurate comparison of studies. International consensus is required. The greatest challenge is to prevent, not only MUO, but obesity in general. A population level approach is required that takes into account known and unknown environmental detrimental effects. More focus on factors influencing early development, including through epigenetic changes, will be important.
